# How to Place a Central Line: Preparing Your Kit & Performing the Procedure

**DOI:** 10.5070/M5.54074

**Published:** 2026-10-31

**Authors:** Christopher San Miguel, Krystin N Miller, Alexander Close, Jennifer Yee

**Affiliations:** * The Ohio State University College of Medicine, Department of Emergency Medicine, Columbus, Ohio

## Abstract

**Audience:**

Senior Medical Students and Interns

**Introduction:**

Central venous catheter (CVC) placement is a critical, high-risk procedure, and it is considered a core component of emergency medicine training.^1^ Iatrogenic complications from improper technique—including arterial dilation, pneumothorax, and line-associated bloodstream infections—can significantly impact patient outcomes.^2^–^7^ Therefore, emergency medicine trainees should ideally demonstrate procedural competence and adherence to safety practices before performing CVC insertion in clinical settings. These videos serve as part of a mastery-based curriculum designed to reinforce standardized, evidence-based techniques and institutional safety protocols for internal jugular CVC placement.^8^ The procedural preparation video focuses on sterile technique, ultrasound setup, and CVC kit preparation, and the procedural performance video focuses on sterile technique, the leapfrog technique for ultrasound-guided needle cannulation, and the safe insertion of the central venous catheter.

**Educational Objectives:**

By the end of these videos, learners will be able to:

1. Identify and gather all necessary materials for internal jugular (IJ) CVC placement.
2. Describe the appropriate positioning of the patient, ultrasound machine, and proceduralist for CVC placement.
3. List common complications of IJ CVC placement.
4. Demonstrate sterile field preparation, including donning personal protective equipment (PPE), placing a sterile drape, and placement of a sterile ultrasound probe cover.
5. Perform a proper pre-procedural time-out including at least two patient identifiers and site verification.
6. Prepare and organize CVC kit components for efficient procedural flow.
7. Identify key anatomical landmarks and optimal patient positioning for right IJ CVC placement using ultrasound guidance.
8. Demonstrate stepwise needle insertion with real-time ultrasound visualization to safely cannulate the internal jugular vein.
9. Verify proper guidewire placement using both short- and long-axis ultrasound views before vessel dilation.
10. Perform safe dilation and catheter advancement using standard procedural technique.
11. Exhibit best-practice behaviors, including port flushing, line securement, and sterile dressing application.
12. Document the procedure and identify post-placement safety steps such as confirmatory chest imaging.

**Educational Methods:**

These videos were watched by emergency medicine-bound, fourth-year medical students as asynchronous study material prior to their mastery-based central venous catheter assessment. Students watched these videos, underwent individual pre-test (baseline) assessments using a 58-item checklist, witnessed a faculty member perform a group demonstration, and then performed deliberate observed practice. The previously published, procedure checklist was developed by a multidisciplinary team of experts, and minimum passing standards were determined using the Mastery-Angoff Technique.

**Research Methods:**

Efficacy of the videos may be inferred through baseline assessment scores, since students were given access to the videos prior to completing their baseline assessment. Utilizing a quasi-experimental design, these scores were compared to previously published data from the previous 2 years in which students completed the baseline assessment without access to the videos.^9^

**Results:**

Ten fourth-year emergency medicine-bound medical students participated in a simulation-based mastery learning procedural course in March 2025. As part of their asynchronous preparation, they watched these two instructional videos: 1. Preparation to Place a Central Venous Cather and 2. Placement of a Central Venous Catheter. Learners were individually assessed in a simulation lab using a 58-item checklist. Pre-test scores ranged from 17 to 89% correct, with a median score of 67%. Previous cohorts from 2022 and 2023 had scores ranging from 19 to 84% with a median score of 43%. After deliberate practice, first post-test scores improved for participants and ranged from 89 to 98% correct.

**Discussion:**

These educational videos were an effective component of a broader simulation-based mastery learning curriculum for central venous catheter placement. These videos provided a standardized introduction to both general and institution-specific patient safety practices related to CVC kit setup and CVC insertion. Although video views were not tracked, learners demonstrated significant improvement between pre- and post-testing, indicating the combined instructional approach was effective. Compared to previous years’ cohorts, the students who had access to the instructional videos scored better on the baseline assessment, though the small number of participants kept this difference from reaching statistical significance. The videos also allowed students to engage with the material at their own pace, reinforcing safety principles and procedural preparation in a consistent and accessible way.

**Topics:**

Procedural Education, Central Venous Catheter, Emergency Medicine.

## USER GUIDE

**Table:** 

List of Resources:	
Abstract	1
User Guide	3
Appendix A: How to Place a Central Line Preparing Your Kit	6
Appendix B: How to Place a Central Line Performing the Procedure	7

**Learner Audience:**

Senior Medical Students and Interns

**Time Required for Implementation:** Watching the videos requires no preparing time. The combined total run time of the two videos is approximately 13 minutes.

**Recommended Number of Learners per Instructor:**

The instructional videos can be widely distributed and reviewed independently by the students. For the in-person portion of the curriculum, including pre-testing, deliberate practice, and post-testing, the instructor to learner ratio is 1:1. These components took approximately 3 hours for each learner.

**Topics:**

Procedural Education, Central Venous Catheter, Emergency Medicine.

**Objectives:**

By the end of these videos, learners will be able to:

1. Identify and gather all necessary materials for IJ CVC placement.
2. Describe the appropriate positioning of the patient, ultrasound machine, and proceduralist for CVC placement.
3. List common complications of IJ CVC placement.
4. Demonstrate sterile field preparation, including donning PPE, placing a sterile drape, and placement of a sterile ultrasound probe cover.
5. Perform a proper pre-procedural time-out including at least two patient identifiers and site verification.
6. Prepare and organize CVC kit components for efficient procedural flow.
7. Identify key anatomical landmarks and optimal patient positioning for right IJ CVC placement using ultrasound guidance.
8. Demonstrate stepwise needle insertion with real-time ultrasound visualization to safely cannulate the internal jugular vein.
9. Verify proper guidewire placement using both short- and long-axis ultrasound views before vessel dilation.
10. Perform safe dilation and catheter advancement using standard procedural technique.
11. Exhibit best-practice behaviors, including port flushing, line securement, and sterile dressing application.
12. Document the procedure and identify post-placement safety steps such as confirmatory chest imaging.

### Linked objectives and methods

Mastery-based education is the only procedural framework that has demonstrated improved downstream patient outcomes. These videos demonstrate safe procedural behaviors utilizing the same kit, ultrasound, and task trainer that learners will engage with during their procedural assessment. Because the videos can be distributed, watched, and reviewed asynchronously, they allow for intentional, efficient asynchronous education to prepare the students for their in-person, simulation-based deliberate practice and assessments. The videos were created using an iPad and the native IOS Camera software. An attending physician who was involved in the creation of the curriculum directed a PGY-3 resident as they prepared for and ultimately completed the CVC placement procedure, using the assessment checklist as a guide. The videos were edited and voice-over was created with the assistance of a freelance editor. Objectives 1–12 are met during the standardized assessment portion of the larger curriculum. The use of simulation may allow for targeted education towards iatrogenic injury prevention prior to actual patient care^8^,^10^,^11^ and has been previously described within a mastery-based framework for emergency medicine orientation^11^ and across a graduate medical education-wide initiative.^8^. In short, students are given a brief introduction to the curriculum and then complete a pre-test assessment during which they complete the procedure while a faculty member assesses their performance against the procedural checklist. The students then all watched an in-person exemplar performance of the procedure completed by a faculty member. They then engaged in individual deliberate practice focused on low scoring areas from the pre-test. Finally, the students were brought back to the simulation lab several days later to complete their post-instructional assessment using the procedural checklist. Students were required to return days later for the post instructional assessment because we felt it better reflected true mastery of the procedures rather than undergoing an assessment immediately following deliberate practice. A Mastery-Angoff standard setting was chosen because it better aligns with simulation-based mastery learning principles compared to a traditional Angoff standard setting. For Mastery-Angoff, raters are asked to consider the performance of a well-prepared resident, compared to the traditional Angoff method in which raters consider a minimally-acceptable performance of a learner for a pass/fail standard. Consequently, the Mastery-Angoff method leads to a higher standard that is often used for patient safety initiatives, such as procedural education.^12^

### Recommended pre-reading for instructor

The instructors are also encouraged to watch the instructional videos in preparation for the in-person sessions. At a minimum, instructors should review the procedural checklist they will be using.

### Learner responsible content

Learners should review these videos prior to a central venous catheter procedure laboratory session or assessment.

### Link to Lecture

- How to Place a Central Line Preparing Your Kit
  ○ https://youtu.be/TBYstxelDBo
- How to Place a Central Line Performing the Procedure
  ○ https://youtu.be/mfHGPx5nMBY

### Results and tips for successful implementation

These videos were implemented in March 2025 with 10 fourth-year medical students pursuing emergency medicine. They were presented to the students as asynchronous pre-work for a simulation-based mastery learning course. Students were assessed on a 58-item checklist before and after a deliberate practice session. Initial scores ranged from 17 to 89% correct with a median score of 67%. Ultimately, first post-test scores improved to a range of 89 to 98% correct.

Previous cohorts from 2022 and 2023, engaged in the same simulation-based mastery curriculum but without the preparatory instructional videos. These cohorts had scores ranging from 19 to 84% with a median score of 43%. A Mann U Whitney test resulted with a U value of 24.5 and *P* value of 0.052. Although we did not meet the 0.05 threshold for statistical significance, we believe this is likely secondary to the limited number of participants. Furthermore, since this is a quasi-experimental design, there was no controlling for confounding variables. Although we are unaware of any significant confounding variables, it is certainly possible that one or more may have contributed to the difference seen between the two groups. Despite this, there is a clear trend to improved baseline scores after students were given access to the instructional videos.

For optimal use, we recommend:

- Embedding the videos within a broader simulation-based curriculum.
- Coordinating with institutional quality or safety teams to ensure alignment with current institutional protocols.
- Using the videos to support deliberate practice and standardize expectations for kit setup.
- Encouraging learners to review the videos shortly before hands-on deliberate practice or clinical performance.

### Technology necessary

The videos may be reviewed on a computer, laptop, or cell phone.

### Assessment

The associated central venous catheter checklist used for mastery-based procedural education has been previously published.^8^

## Figures and Tables

**Figure:**
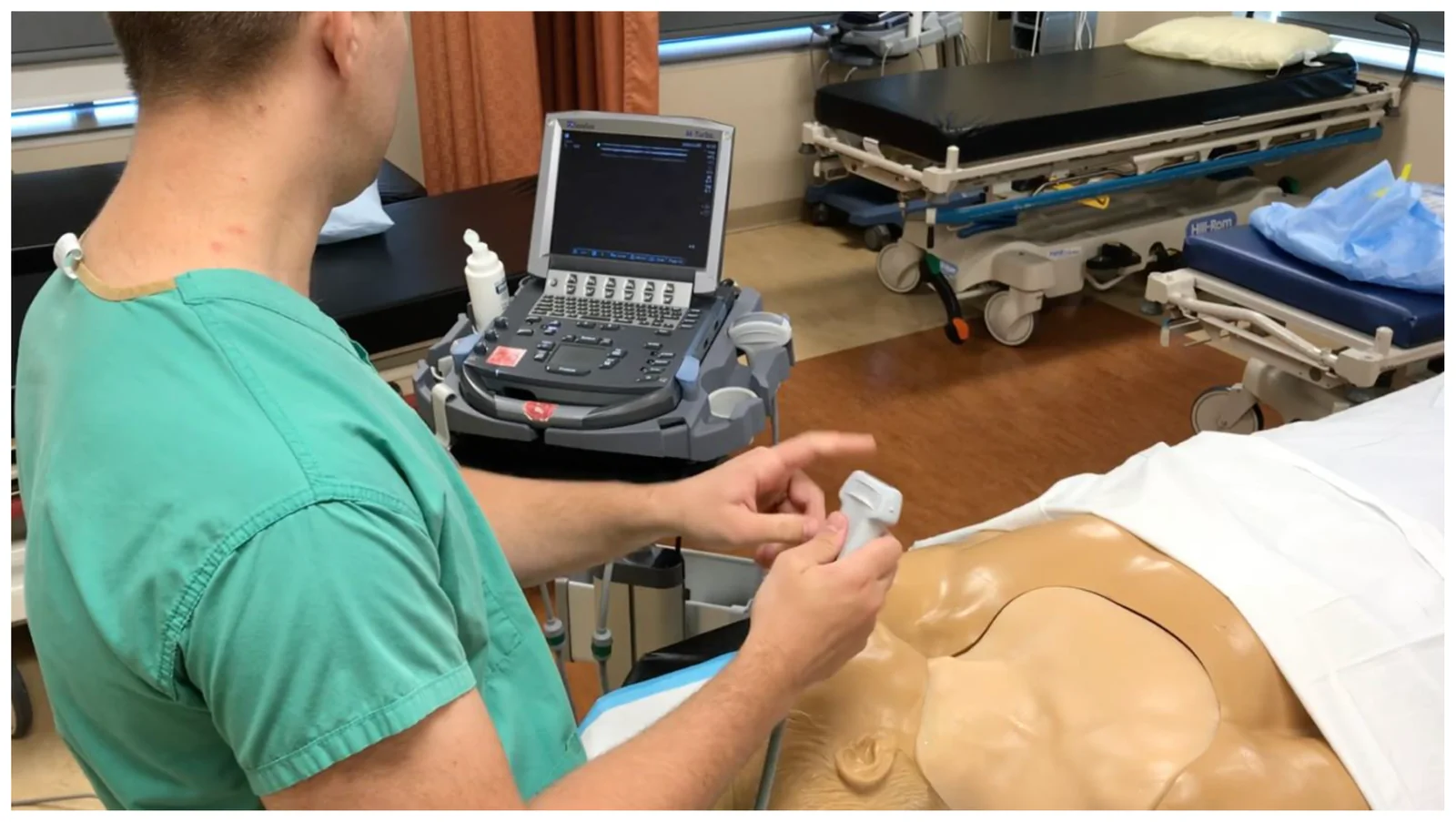
Please see associated video file Video Link: https://youtu.be/TBYstxelDBo

**Figure:**
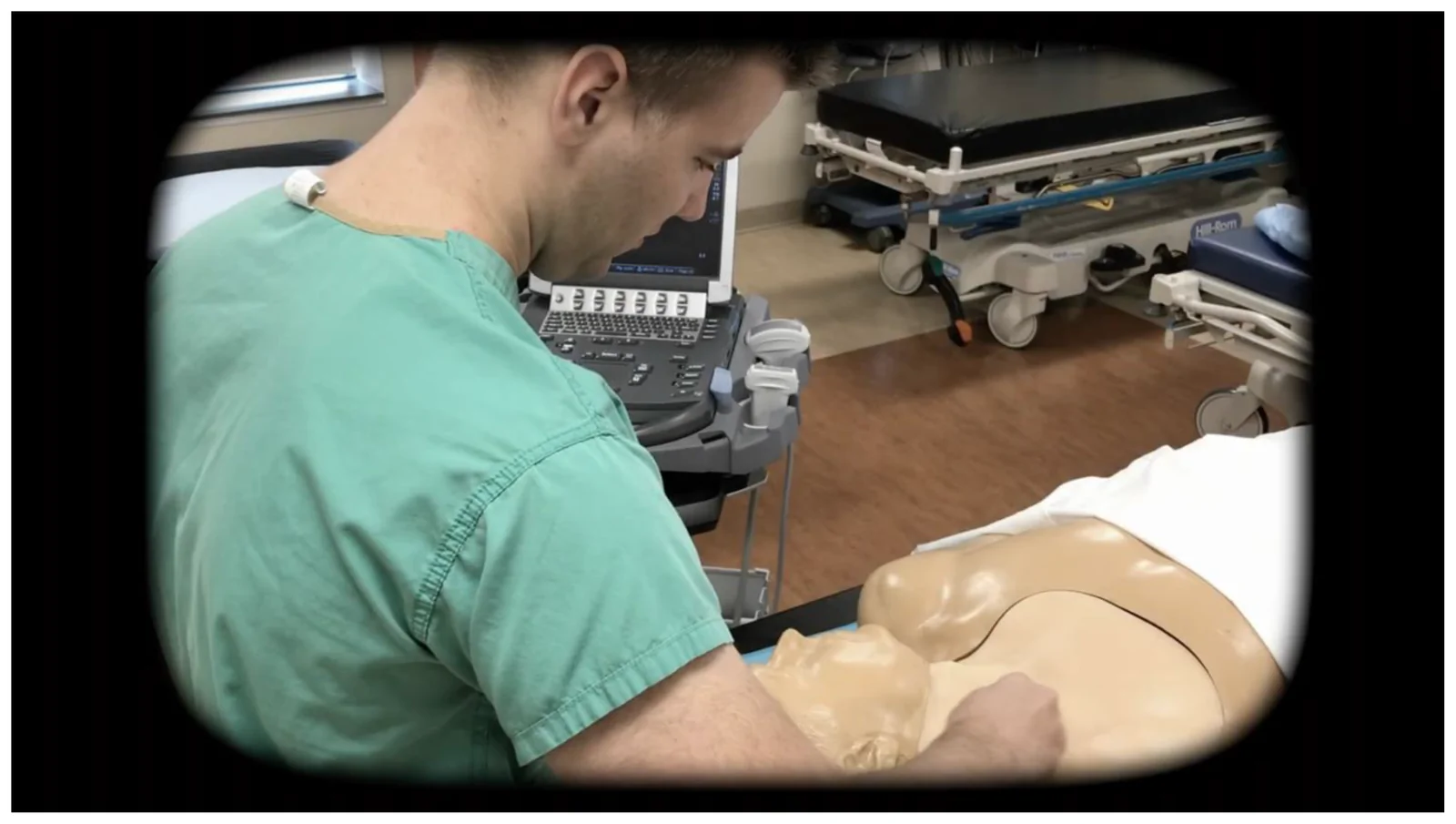
Please see associated video file Video Link: https://youtu.be/mfHGPx5nMBY
